# Editorial: Advances in biofabrication for skin regeneration

**DOI:** 10.3389/fbioe.2023.1223797

**Published:** 2023-05-30

**Authors:** Juliana R. Dias, Alessia Longoni, Ana L. Oliveira

**Affiliations:** ^1^ Centre for Rapid and Sustainable Product Development, Instituto Politécnico de Leiria, Marinha Grande, Portugal; ^2^ Department of Orthopaedic Surgery and Musculoskeletal Medicine Centre for Bioengineering and Nanomedicine, University of Otago Christchurch, Christchurch, New Zealand; ^3^ CBQF—Centro de Biotecnologia e Química Fina—Laboratório Associado, Escola Superior de Biotecnologia, Universidade Católica Portuguesa, Porto, Portugal

**Keywords:** TE-based skin substitutes, wound healing, hybrid structures, *in situ* approaches, controlled drug delivery

The development of TE-based skin substitutes is one of the most promising approaches to achieve efficient skin regeneration in the case of acute and chronic wounds (Ferreira et al., 2021; Dias et al., 2022). In fact, biofabrication strategies have demonstrated that is possible to produce advanced structures that mimic the morphology, properties, and functions of skin ECM, promoting faster and reliable wound healing (Dias et al., 2020).

This Research Topic includes interesting and innovative research papers that can lead to advances in the field of TE-based skin substitute development. Immediately after a skin injury occurs, a cascade of events begin, with haemostasis being the first. Thus, bleeding control and wound protection is crucial. In the study by Zhang et al., a carboxymethyl cellulose/carboxymethyl chitosan-polydopamine (CMC/CMCS-PDA) sponge exhibiting *in vivo* skin haemostasis and repair properties was developed. Additionally, it presented good antibacterial and antioxidant properties. The second research paper included in this Research Topic is the study by Wang et al., where 3D-printed egg white hydrogels promoting adipose-derived stem cells (ASCs) adhesion and proliferation without cytotoxicity were developed. Furthermore, *in vivo* results showed the ability of these ASC-seeded hydrogels to accelerate wound healing through the enhancement of fibroblast proliferation, angiogenesis, and collagen rearrangement in the wound bed. In another interesting clinical study, Xue et al. demonstrated the effectiveness of injectable platelet-rich fibrin (i-PRF), produced via a simple two-centrifugation method combined with vacuum sealing drainage, in reducing wound inflammation and promoting scar formation in patients with chronic refractory wounds (CRW).

Another important aspect that should be considered when aiming to regenerate skin is wound infection, as the presence of a bacterial infection could delay the healing process. Considering that antimicrobial resistance is one of the greatest global public health challenges of our time, the search for alternatives to antibiotics is crucial for wound management. Based on this, this Research Topic includes a review article by Alipoor et al., in which the bactericidal activity of hyaluronic acid (HA) was detailed together with a summary of the HA structure, its production and properties, and its various platforms as a carrier in drug delivery.

Overall, the reports presented in this Research Topic highlight the importance of advanced approaches to treat wounds, to promote haemostasis, and to develop new alternatives to synthetic antibiotics. As Research Topic Editors, we are deeply grateful to all the authors for their outstanding-quality research and to all the reviewers for their critical evaluations of the manuscripts.
